# Parkin Mutation Affects Clock Gene-Dependent Energy Metabolism

**DOI:** 10.3390/ijms20112772

**Published:** 2019-06-05

**Authors:** Consiglia Pacelli, Giovannina Rotundo, Lucia Lecce, Marta Menga, Eris Bidollari, Rosella Scrima, Olga Cela, Claudia Piccoli, Tiziana Cocco, Angelo Luigi Vescovi, Gianluigi Mazzoccoli, Jessica Rosati, Nazzareno Capitanio

**Affiliations:** 1 Department of Clinical and Experimental Medicine, University of Foggia, 71122 Foggia, Italy; lucia.lecce@unifg.it (L.L.); marta.menga@unifg.it (M.M.); rosella.scrima@unifg.it (R.S.); olga.cela@unifg.it (O.C.); claudia.piccoli@unifg.it (C.P.); 2 Cell Reprogramming Unit, Fondazione IRCCS Casa Sollievo della Sofferenza, 71013 San Giovanni Rotondo (FG), Italy; g.rotundo@css-mendel.it (G.R.); e.bidollari@css-mendel.it (E.B.); j.rosati@css-mendel.it (J.R.); 3 Laboratory of Pre-Clinical and Translational Research, IRCCS-CROB, Referral Cancer Center of Basilicata, 85028 Rionero in Vulture (PZ), Italy; 4 Department of Basic Medical Sciences, Neurosciences and Sensory Organs, University of Bari “Aldo Moro”, 70124 Bari, Italy; tizianamaria.cocco@uniba.it; 5 Department of Biotechnology and Biosciences, Bicocca University of Milan, 20126 Milan, Italy; vescovia@gmail.com; 6 Division of Internal Medicine and Chronobiology Unit, Fondazione IRCCS Casa Sollievo della Sofferenza, 71013 San Giovanni Rotondo (FG), Italy; g.mazzoccoli@operapadrepio.it

**Keywords:** parkin, mitochondria, circadian clock, Parkinson´s disease, neurodegeneration, phenotypic reprogramming

## Abstract

Growing evidence highlights a tight connection between circadian rhythms, molecular clockworks, and mitochondrial function. In particular, mitochondrial quality control and bioenergetics have been proven to undergo circadian oscillations driven by core clock genes. Parkinson’s disease (PD) is a chronic neurodegenerative disease characterized by a selective loss of dopaminergic neurons. Almost half of the autosomal recessive forms of juvenile parkinsonism have been associated with mutations in the *PARK2* gene coding for parkin, shown to be involved in mitophagy-mediated mitochondrial quality control. The aim of this study was to investigate, in fibroblasts from genetic PD patients carrying parkin mutations, the interplay between mitochondrial bioenergetics and the cell autonomous circadian clock. Using two different in vitro synchronization protocols, we demonstrated that normal fibroblasts displayed rhythmic oscillations of both mitochondrial respiration and glycolytic activity. Conversely, in fibroblasts obtained from PD patients, a severe damping of the bioenergetic oscillatory patterns was observed. Analysis of the core clock genes showed deregulation of their expression patterns in PD fibroblasts, which was confirmed in induced pluripotent stem cells (iPSCs) and induced neural stem cells (iNSCs) derived thereof. The results from this study support a reciprocal interplay between the clockwork machinery and mitochondrial energy metabolism, point to a parkin-dependent mechanism of regulation, and unveil a hitherto unappreciated level of complexity in the pathophysiology of PD and eventually other neurodegenerative diseases.

## 1. Introduction

Parkinson’s disease (PD) is a chronic progressive neurodegenerative movement disorder characterized by the selective loss of dopaminergic neurons in the substantia nigra pars compacta. While most cases of PD occur sporadically as the result of many different environmental factors, several gene products have been identified as responsible for Mendelian forms of PD, both autosomal and recessive [1]. Since the discovery of mutations in the parkin encoding gene (PARK2) as a cause of autosomal recessive juvenile parkinsonism [2] almost half of all PD cases have been associated with mutations in this gene. Parkin is a multifunctional E3 ubiquitin ligase, which mediates ubiquitination of various target proteins [3]. Several studies on parkin-null animal models strongly suggest an important role of the parkin encoding gene for the preservation of mitochondrial function. Despite having only mild deficits, parkin knockout mice exhibit mitochondrial dysfunction and oxidative damage [4,5] and *Drosophila* parkin-null mutants display mitochondrial pathology and apoptotic muscle degeneration [6,7]. Functional assays in leukocytes [8] as well as fibroblasts of patients with parkin mutations consistently show mitochondrial impairment [9,10,11]. Recently, increasing experimental evidence revealed that parkin interacts with PTEN-induced kinase 1 (PINK1) in a common pathway regulating mitochondrial dynamics [12] and mitophagy [13]. Furthermore, parkin has emerged as an important factor in the mitochondrial quality control mechanisms [14,15] and in Ca^2+^-dependent homeostasis [16,17,18].

Besides mitochondrial dysfunction, a key role for deranged circadian pathways is recently emerging in PD physiopathology, responsible of non-motor PD symptoms [19]. Increasing evidence has shown reciprocal interactions between dopaminergic system and circadian pathways: tyrosine hydroxylase, the rate-limiting enzyme for dopamine (DA) synthesis, is regulated by the circadian locomotor cycle kaput (*CLOCK*) gene [20], and DA itself can upregulate the transcriptional activity of the CLOCK/BMAL1 complex through enhancing the recruitment and phosphorylation of the transcriptional coactivator cAMP-responsive element-binding protein [21]. Alterations in circadian rhythms have been reported in several PD animal models, both genetic [22] and toxin-induced PD models [23,24,25], as well as in PD patients [26,27,28].

Interestingly, in the past few years mounting evidence has highlighted the tight correlation between circadian rhythms and metabolism. In particular, a new interlocked transcriptional-enzymatic feedback loop controlling the molecular interplay between cellular bioenergetics and the molecular clockwork has been highlighted [29,30]. Furthermore, in mouse liver it has been shown that mitochondrial biogenesis and dynamics (notably fission–fusion cycles and mitophagy) are transcriptionally driven by the circadian regulator Bmal1 and exhibit metabolic rhythms in sync with nychthemeral bioenergetics demands [31]. The evidence of alterations of these processes in parkin-null fibroblasts make these cells suitable to study the interplay of mitochondrial function and circadian pathways. Our results show that mitochondrial alterations in PD fibroblasts are linked to the deregulation of the molecular clockwork, thus providing evidence of the involvement of additional players in PD onset and progression.

## 2. Results

### 2.1. Synchronized Fibroblasts Exhibit Autonomous Oscillatory Mitochondrial Respiration and Glycolytic Activity

**Figure 1:**
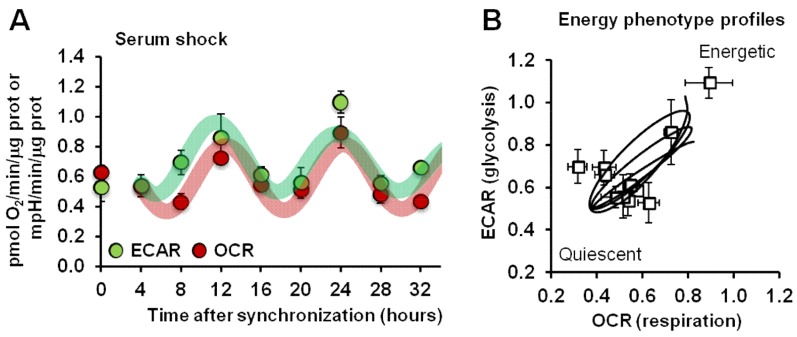
Analysis of mitochondrial respiration and glycolysis in serum shocked synchronized normal human dermal fibroblast (NHDF) cells. (**A**) The oxygen consumption rate (OCR) and extracellular acidification rate (ECAR) were assessed by a Seahorse Analyzer in intact NHDFs every four hours post-synchronization for 32 h under resting conditions. Data are expressed as pmol O_2_/min/μg protein for OCR and mpH/min/μg protein for ECAR. See Materials and Methods for experimental details. (**B**) Energy map obtained plotting the ECAR and OCR values obtained in (**A**).

Commercially available normal human dermal fibroblasts (NHDFs) were subjected to a well-established synchronization protocol consisting in serum shock (treatment with high concentrations of serum) to reset the clockwork machinery, thus allowing homogeneous synchronization of clock gene expression in cultured cells once the repressing condition is released [32]. The oxygen consumption rate (OCR) and extracellular acidification rate (ECAR), indicators of mitochondrial respiratory activity and glycolytic activity, respectively, were assessed using a Seahorse Analyzer in intact NHDFs every 4 h for 34 h post-synchronization. Figure 1A shows that following serum shock synchronization, NHDFs displayed a rhythmic activity of the basal OCR, which peaked 12 and 24 h after synchronization. Notably, when synchronized cells were assayed for glycolytic activity, a time-dependent profile of basal ECAR superimposable to that of OCR was observed. The results obtained in synchronized NHDFs indicated that the two major energy-producing metabolic pathways co-occurred and did not compensate each other, pointing to alternating phases in the overall cell bioenergetics (Figure 1B).

Similar results were obtained when NHDFs were synchronized by a different protocol consisting of a short-term treatment with forskolin [33], thus implying that cultured fibroblasts are endowed with an autonomous metabolic rhythmic activity elicited independently of the utilized synchronization protocol (Supplementary Figure S1).

The above described metabolic rhythmic activity was also shown in different types of synchronized cell lines and proved to be linked to the expression of the core clock gene *BMAL1* [30].

### 2.2. Energy Metabolism in Patient Fibroblasts with the Parkin Mutation

Next, we analyzed the metabolic fluxes in dermal fibroblasts isolated from two unrelated patients affected by early-onset Parkinson’s disease (PD) and carrying the mutations in *PARK2* previously described [10,11]. In particular, fibroblasts from patient 1 (hereinafter defined as P1) carried a heterozygous del exon2–3/del exon3 mutation, and fibroblasts from patient 2 (hereinafter defined as P2) carried heterozygous del exon7–9/Glu409X mutation; these mutations led to the complete absence of the parkin protein in both patients [10,11]. Dermal fibroblasts isolated from two healthy subjects were used as controls (CTRL).

**Figure 2:**
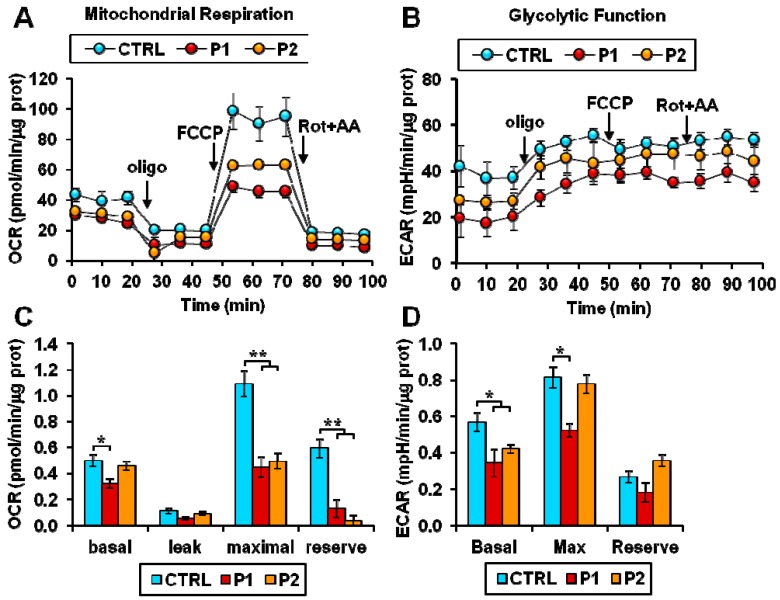
Energy metabolism in patient fibroblasts with the parkin mutation. (**A**,**B**). Representative Seahorse XF trace of OCR (**A**) and ECAR (**B**) performed in normal (CTRL) and Parkinson’s disease (PD) patient (P1, P2) fibroblasts. For each trace, after a baseline reading, oligomycin A (oligo), FCCP, and rotenone + antimycin A (Rot + AA) were added as indicated by the arrows. See Materials and Methods for experimental details. (**C**,**D**) The bar graphs display the data obtained from experiments shown in (**A**,**B**). (**C**) Basal: resting OCR; leak: OCR measured in the presence of oligomycin; maximal: OCR measured in the presence of FCCP; reserve: difference between maximal and basal respiration. The OCR values were corrected for the residual OCR measured in the presence of rotenone and antimycin A. (**D**) Basal: resting ECAR; Max: ECAR measured in the presence of oligomycin and FCCP; Reserve: difference between maximal and basal glycolysis. Data show the mean ± SEM of four independent experiments performed in triplicate. Significance was calculated with the Student’s *t*-test; * *p* < 0.01, ** *p* <0.005 vs. CTRL cells.

Figure 2A,B illustrate representative recordings of the OCR and ECAR under different conditions in control and patient fibroblasts as assessed by the Seahorse Analyzer. Under basal conditions a statistically significant reduction in OCR was observed in P1 fibroblasts. Addition of the H^+^-ATP-synthase inhibitor oligomycin depressed the OCR, which was controlled by the passive proton back-leak. Further addition of the protonophore uncoupler FCCP resulted in increased OCR, which was no longer inhibited by the membrane potential; however, the maximal OCR resulted significantly higher than under basal conditions only in control fibroblasts (Figure 2A,C). Consequently, the difference between the maximal and the basal OCRs, which is taken as a measure of the reserve respiratory capacity (or spare respiratory capacity) was dramatically reduced in both P1 and P2 fibroblasts (Figure 2C). The ECAR, linked to the glycolytic flux, resulted significantly lower in both patients’ fibroblasts and in P1 under conditions maximizing the glycolytic flux (i.e., in the presence of oligomycin) (Figure 2B,D) thereby demonstrating a lack of metabolic switch to compensate a respiratory deficit.

Taken together, these results revealed an overall bioenergetics impairment in fibroblasts from PD patients, which was particularly evident when cells were assessed under stressed conditions.

**Figure 3:**
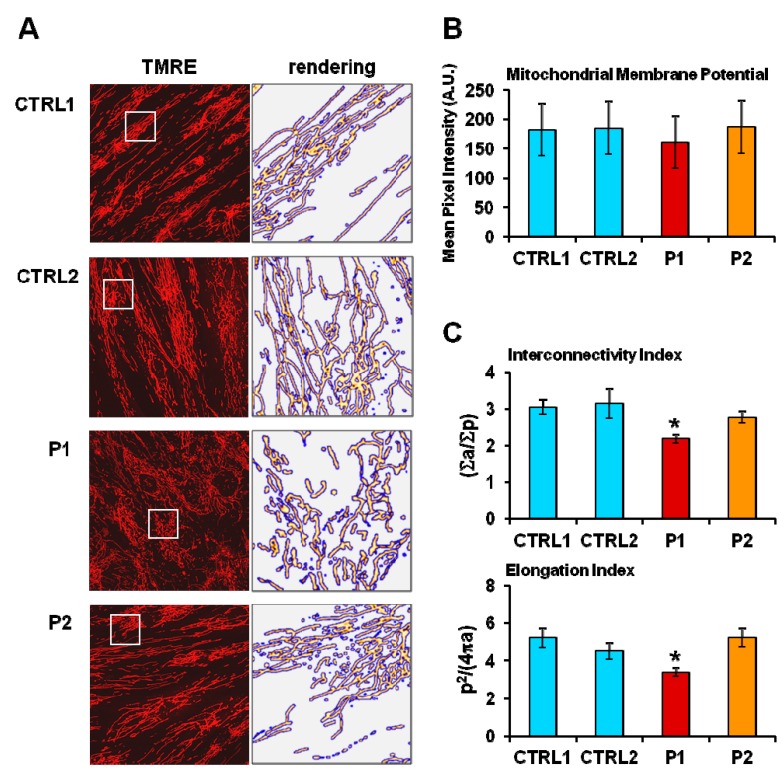
(**A**) Representative confocal images of mitochondrial membrane potential and morphology in two different normal fibroblasts (CTRL) and in PD patient-derived (P1 and P2) fibroblasts. Cells were treated with the fluorescent probe tetramethylrhodamine ethyl ester (TMRE) as described in the Materials and Methods: the objective magnification was 60×. The panels on the right are enlargements of details shown as white square displaying the mitochondrial network after removal of the background and rendering of the TMRE-related pixel intensities with false color images by ImageJ software. (**B**) Statistical analysis of the TMRE-related mean pixel intensity with the bars indicating the values averaged from 8 to 10 different optical fields from three independent experiments for each sample (± SD). (**C**) Morphometric analysis of the TMRE-stained mitochondria showing the interconnectivity index (∑a/∑p) and the elongation index (p^2^/4πa) computed as described in the Materials and Methods. The results shown are values (± SEM) averaged from a total of 15 different optical fields from three independent measurements for each sample; significance was calculated with Student’s *t*-test * *p* < 0.01 vs. CTRL 1.

Morpho-functional analysis of the mitochondrial compartment by confocal microscopy using the specific mitochondrial probe tetramethylrhodamine ethyl ester (TMRE) revealed that, though not showing apparent changes in the mitochondrial membrane potential, P1 fibroblasts exhibited more fragmented mitochondria, as demonstrated by a decrease in interconnectivity index and elongation in P1, showing the most severe bioenergetic impairment (Figure 3).

### 2.3. Circadian Clock-Dependent Energy Metabolism in Patients’ Fibroblasts with Mutated Parkin

Several studies have indicated that the biological clock orchestrates mitochondrial metabolism [34,35] and that, reciprocally, mitochondria-related functions modulate the expression of clock genes [29,36]. The energy producing capacity of mitochondria is strongly related to their abundance and morphology, which depend on biogenetic, mitophagic, and dynamic processes [37]. The PINK1/parkin-related pathway is a well-established sequential process that coordinates several mitochondrial quality control facets [38].

**Figure 4:**
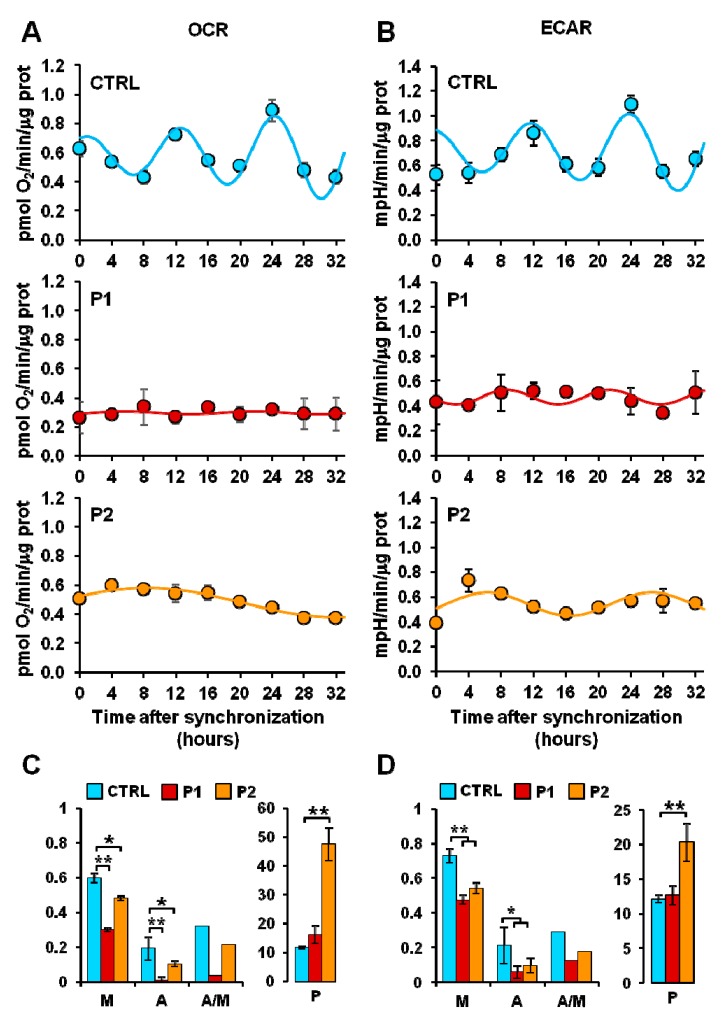
Measurement of the mitochondrial respiratory and glycolytic activity in serum shock-synchronized intact fibroblasts. (**A**) Resting oxygen consumption rate (OCR) and (**B**) basal extracellular acidification rate (ECAR) in serum shock-synchronized normal fibroblasts (CTRL) and PD patient-derived fibroblasts (P1, P2)—the time-course data points were best fitted with the COSINOR function. (**C**,**D**) The histograms display the mesor (M), the amplitude (A), and the A/M ratio for respiration (**C**) and glycolysis (**D**) of the best-fitting COSINOR. See Materials and Methods for details. Data are mean ± SEM of two independent experiments performed in triplicate. Statistical significance was calculated with Student’s *t*-test * *p* < 0.01, **p < 0.005 vs. CTRL.

**Figure 5:**
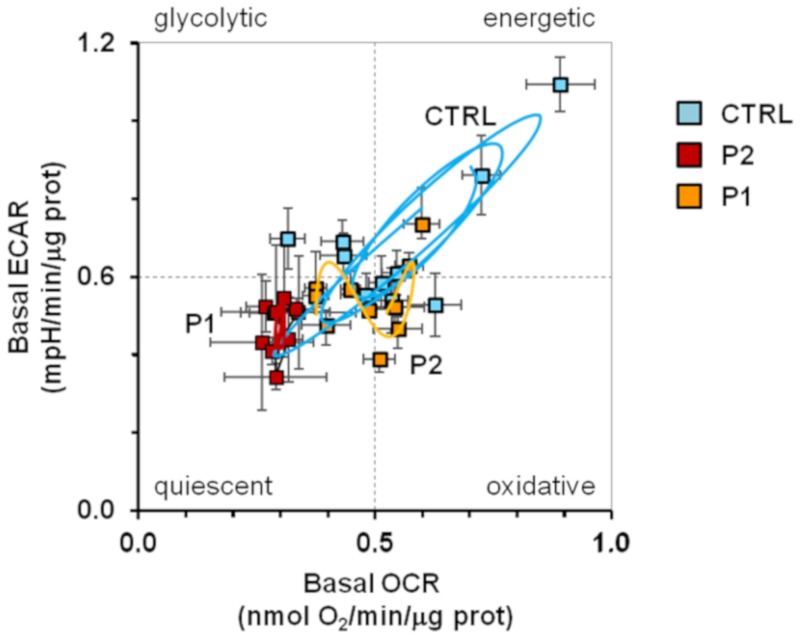
Bioenergetic profile in synchronized intact fibroblasts. The graph displays the energy map obtained plotting the resting extracellular acidification rate (ECAR) and oxygen consumption rate (OCR) values obtained from data shown in Figure 4 for normal fibroblasts (CTRL, light blue square), and PD patient fibroblasts (P1, red square; P2, orange square); the continuous lines are computed merging the best-fitting COSINOR curves for OCR and ECAR.

In keeping with this notion we pondered whether alterations in the mitochondrial quality control, such as those observed in *PARK2* mutated fibroblasts, could affect the circadian rhythmicity of mitochondrial respiration. To this aim, the fibroblasts from healthy subjects and PD-affected patients were synchronized by serum shock in vitro and their metabolic fluxes assessed every 4 h for 32 h. Figure 4 shows that like the commercial NHDFs (in Figure 1), dermal fibroblasts isolated from healthy subjects showed similar rhythmic and in-phase oscillations of both basal OCR and ECAR. Conversely, both the P1 and P2 fibroblasts displayed a dramatic damping of the rhythmic metabolic fluxes. In particular, when the experimental time-points were fitted with the COSINOR function [39] a significant reduction of the mesor and amplitude parameters in OCR and ECAR (as well as of the amplitude/mesor ratio) was observed both in P1 and P2 as compared with CTRL fibroblasts. The period was significantly enhanced only in P2 cells. Consequently, the bioenergetic profiles of the control and PD patients fibroblasts resulted in distinct patterns (Figure 5); while the CTRL fibroblasts clearly oscillated between energetic and quiescent state, the metabolic fluxes of P1 and P2 fibroblasts remained confined in the low-energetic region of the bioenergetic profile, with P1 fibroblasts resulting more dramatically affected. Importantly, the above reported results, which refer to the basal OCR and ECAR, were also observed when the metabolic fluxes were maximally stimulated upon addition of FCCP or oligomycin, respectively (Supplementary Figure S2A). Moreover, the results were independent on the protocol of synchronization used; indeed similarly marked alterations of the rhythmic metabolic fluxes were attained in PD patient cells synchronized by forskolin pre-treatment (Supplementary Figure S2B).

### 2.4. Circadian Expression of Genes Involved in Mitochondrial Quality Control

As previously reported, mitochondrial dynamics, biogenesis, and mitophagy are transcriptionally targeted by the circadian regulator BMAL1 and exhibit a metabolic rhythm in sync with diurnal and nocturnal bioenergetic demands [31,40]. Additionally, in cultured fibroblasts it has been shown that the morphology of the mitochondrial network exhibits Drp1-dependent circadian rhythmicity, changing from a tubular to a fragmented mitochondrial network and matching the rhythmicity of ATP content and oxidative phosphorylation [41].

**Figure 6:**
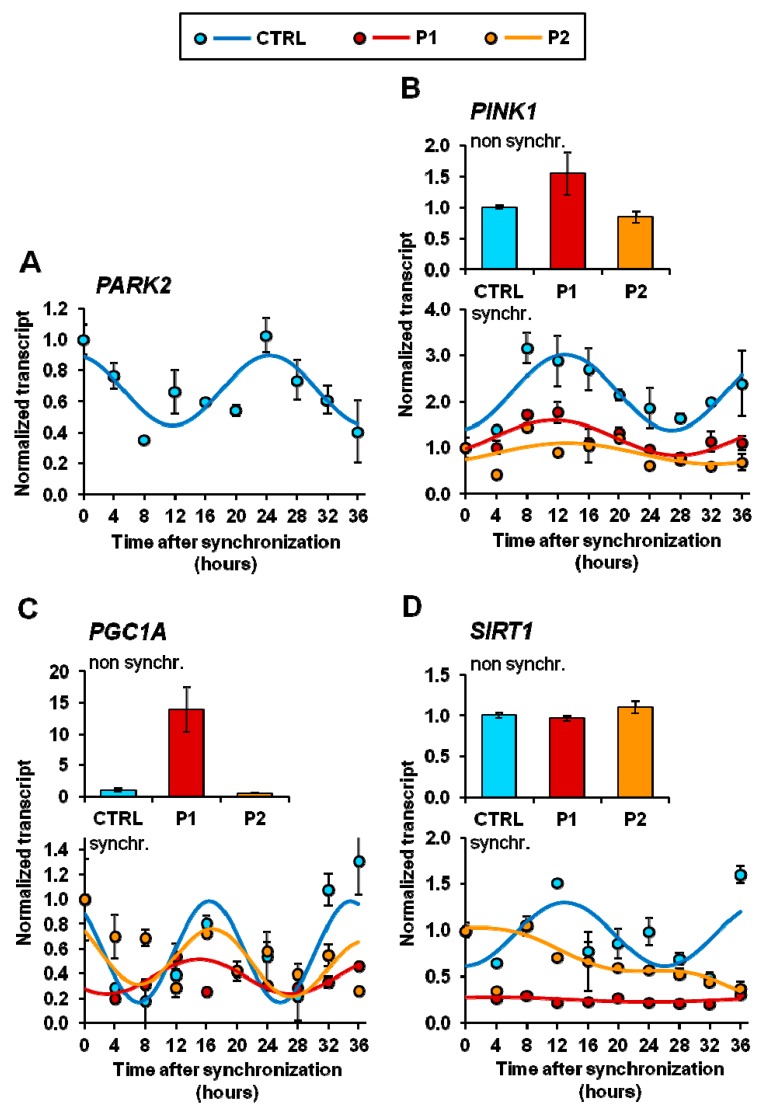
Transcription levels of *PARK2*, *PINK1*, *PGC1A*, and *SIRT1* in normal (CTRL) and PD patient-derived (P1, P2) fibroblasts. The mRNA isolated from non-synchronized and serum shocked-synchronized fibroblasts were subjected to qRT-PCR analysis and the values of *PARK2* (**A**), *PINK1* (**B**), *PGC1A* (**C**), and *SIRT1* (**D**) normalized to the housekeeping gene GAPDH. Data from non-synchronized cells were expressed as fold changes compared to CTRL cells. Data from synchronized cells are expressed as fold change compared to t = 0 and best fit with the COSINOR function. All the values shown are means ± SEM of two independent experiments performed in triplicate under each condition. See Materials and Methods for experimental details.

Given the recognized role played by *PARK6/PARK2* genes in regulating many aspects of mitochondrial physiology, we decided to assess their transcript level in synchronized CTRL- and PD fibroblasts; the results of q-RT-PCR are illustrated in Figure 6. Transcription of both *PARK2* (coding for parkin) and *PARK6* (coding for PINK1) resulted in an oscillation in serum shock-synchronized CTRL fibroblasts for a period close to 24 h, though with a different phase (Figure 6A,B). Notably, even if the transcript level of *PINK1* was not significantly different from that of CTRL fibroblasts under non-synchronized conditions, a marked damping of its oscillation amplitude was observed both in P1 and P2 fibroblasts. Next, we measured the expression of *PGC1A* (coding for peroxisome proliferator-activated receptor (PPAR)-γ coactivator(PGC)-1α) known to be involved in mitochondrial biogenesis as well as in integrating biological clock and energy metabolism [40]. Confirming our previous observations [10], the basal level of the *PGC1A* transcript resulted strongly up-regulated in non-synchronized P1 fibroblasts as compared with CTRL fibroblasts. However, following synchronization the *PGC1A* transcripts, normalized to the zero synchronization-time, resulted in an inhibited oscillatory profile as compared with that in CRTL fibroblasts (Figure 6C). The basal level of the *PGC1A* transcript resulted strongly up-regulated in P1, confirming our previous observations [10], but its normalized circadian oscillation resulted inhibited (Figure 6C). As the activity of *PGC1A* is post-translationally controlled by the SIRT1 deacetylase, which is transcriptionally controlled by clock genes [42,43] and interplays with PERIOD (PER) 2, a circadian core protein [44,45], we further investigated its transcription features. Figure 6D shows that in spite of the apparent absence of effects on the transcription of *SIRT1* under basal conditions, synchronized P1 and P2 cells displayed a marked deregulation of the circadian rhythmicity clearly observed in CTRL fibroblasts.

Taken together the above-reported results clearly show that fibroblasts harboring mutated parkin are hallmarked by severe deregulation of a number of mitochondria-related functions, not particularly evident when assessed under basal conditions but dramatically manifested upon cell synchronization settings.

### 2.5. Circadian Clock-Gene Expression in Patient Fibroblasts is Deregulated in Parkin-Null Fibrolasts

Prompted by the above reported observations we investigated the expression level of the circadian core-clock genes in normal and PD patients’ fibroblasts. First we assessed the transcript level under non-synchronized cell culture conditions. As shown in Supplementary Figure S3, compared with CTRL samples, P1 fibroblasts showed statistically significant up-regulation in the expression of *CLOCK*, *CRY1*, *CRY2*, and *NRD1* and down-regulation of *PER2*. Conversely P2 fibroblasts showed down-regulation in the expression of *PER3*. No changes were observed in the expression of *BMAL1* and *PER1* between CTRL and patients’ fibroblasts.

**Figure 7:**
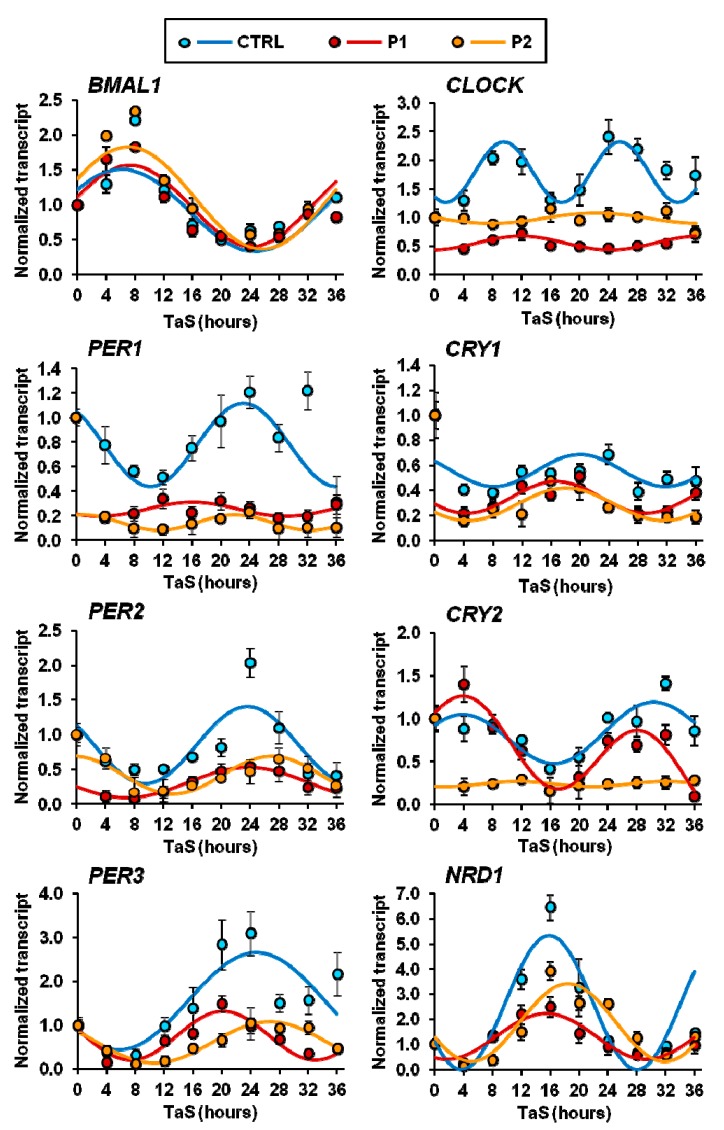
Transcription levels of core clock genes in serum-shocked synchronized normal (CTRL) and PD-derived (P1, P2) fibroblasts. mRNA levels of the indicated genes were assessed by qRT-PCR analysis and normalized to the housekeeping gene *GAPDH*. The time-points are expressed as fold-changes compared to time after synchronization (TaS) = 0 and best fit with the COSINOR function. The values shown are means ± SEM of two independent experiments performed in triplicate under each condition. See Materials and Methods for experimental details.

Next we measured the expression of the core clock genes in serum shock-synchronized cells and analyzed the time-dependent profiles by COSINOR fitting. As shown in Figure 7, the synchronized CTRL fibroblasts displayed oscillatory expression of all the core-clock gene for a period close to 24 h and relative phase shift as expected for the feed-back repressive limb of the transcriptional core-clock machinery [46]. Conversely, with the exception of *BMAL1*, the expression of all the other clock genes resulted deregulated in synchronized P1 and P2 fibroblasts. In particular, the major alterations were observed for *CLOCK* and *PER1* in P1 and P2 and for *CRY2* in P2; in these cases, the rhythmic expression was dramatically damped if not suppressed. However, for *CRY1*, *PER2*, *PER3,* and *NR1D1* significant changes in the amplitude of the oscillatory phases were observed in the fibroblasts derived from both PD patients.

### 2.6. Core Clock Genes Expression in Normal and PD Patient Fibroblast-Derived Induced Pluripotent Stem Cells and Neurospheres

Recent progress in induced pluripotent stem cell (iPSC) technology is providing a remarkable alternative for the study of human brain diseases through the production of human neural cells derived directly from patients with diverse neurological diseases. From this perspective we first derived iPSCs from the skin fibroblasts of the PD patient P1 and then we generated induced neural stem cells (iNSCs) as neurospheres from iPSC-derived embryoid bodies [47]; iPSCs and iNSCs generated from normal fibroblasts were used as control samples. The flowcharts of the procedure are detailed in the Material and Method section and illustrated in the Supplementary Figure S4A–G and Figure S5.

**Figure 8:**
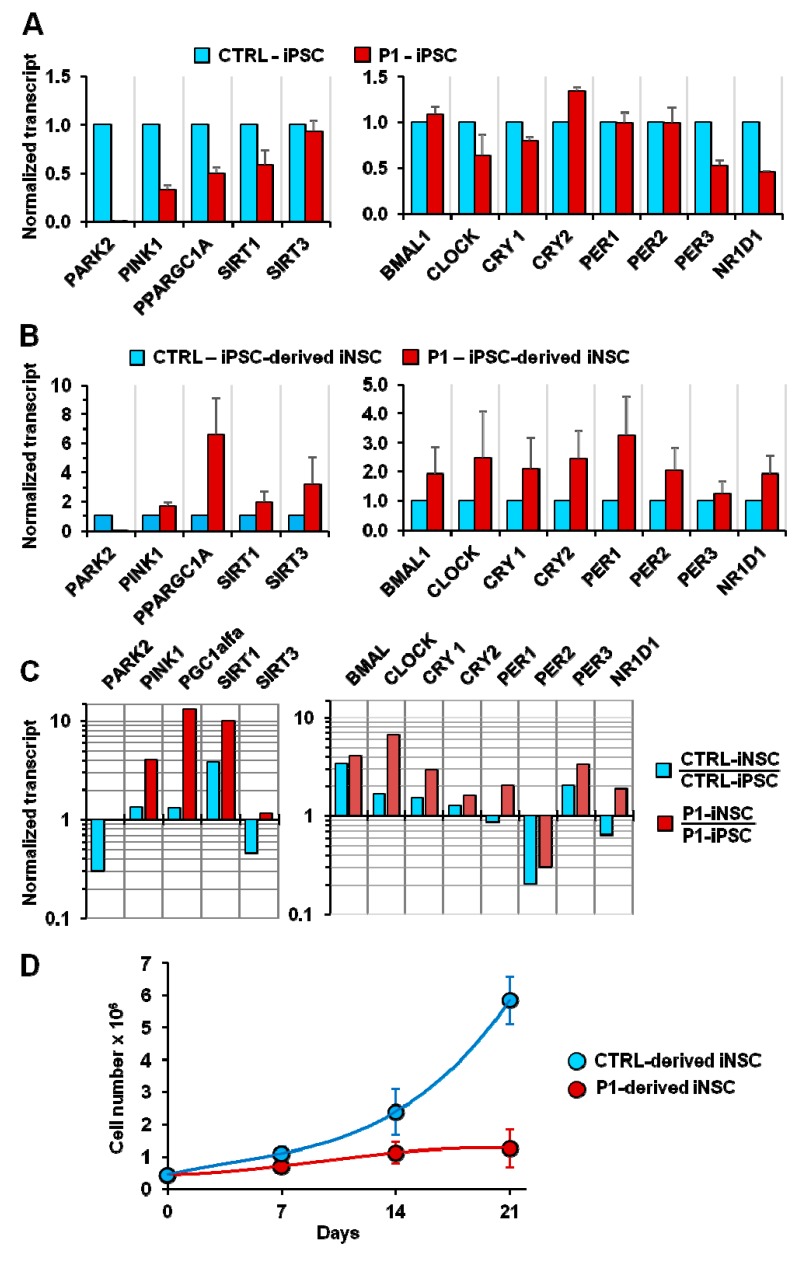
Transcription levels of mitochondrial quality control/biogenesis-related and core clock genes in induced pluripotent stem cells (iPSCs) (**A**) and in induced neural stem cells (iNSCs) (**B**). iPSCs and iNSCs were generated from normal (CTRL) and PD patient-derived (P1) fibroblasts as detailed in the Materials and Methods. The transcript levels of the indicated genes in P1-derived iPSCs/iNSCs were normalized to those of the CTRL-derived iPSCs/iNSCs. (**C**) Fold changes in the expression of the indicated genes in iNSCs as compared with the same in iPSCs. (**D**) Comparative growth curves of neurospheres generated from CTRL and P1 iPSCs. The bar values shown in (**A**) and (**B**) are means ± SEM of two independent measurements carried out in technical triplicate for each gene; the values in (**D**) are means ± SEM of three independent assays.

The mutation in parkin apparently did not affect iPSC nor iNSC generation, and were successively utilized to validate during neurogenesis and in differentiated dopaminergic neurons the altered mitochondrial physiology/biological clock interplay observed in parkin-mutated fibroblasts. As a first line of investigation we assessed the expression level of genes involved in mitochondrial quality control and biogenesis as well as in the molecular clockwork in non-synchronized conditions. Figure 8A shows that when compared with normal fibroblasts-derived iPSC, those derived from P1 fibroblasts displayed statistically significant reductions in the expression of *PINK1*, *PGC1A*, and *SIRT1*; likewise P1 iPSCs showed down-regulation of *CLOCK*, *PER3* and *NR1D1* (coding for REV-ERB-α) expression. Intriguingly, when the same analysis was carried out with iNSCs, the transcript levels of all the considered genes were up-regulated in P1-derived iNSCs (Figure 8B). When the expression of the core clock genes of iNSCs was normalized to those of iPSCs, the transcript levels of *BMAL1*, *CLOCK*, *CRY1*, *CRY2*, and *PER3* were up-regulated in both CTRL and P1 iNSCs, though to a relatively larger extent in P1-derived iNSCs; *PER2* was down-regulated in both CTRL and P1 iNSCs. Conversely, *PER1* and *NR1D1* were up-regulated in P1-iNSCs but down-regulated in CTRL-iNSCs (Figure 8C). Taken together the above-reported transcriptional analysis suggests that parkin mutation caused a qualitative and quantitative deregulation in the co-ordinated expression of genes involved in mitochondrial dynamics and in the clockwork machinery both in iPSCs and iNSCs and following neurogenesis. Of note, the growth rate of P1-derived iNSCs resulted strongly decreased as compared with CTRL iNSCs (Figure 8D), suggesting defective cell bioenergetics and recapitulating what was previously observed for P1 fibroblasts [10].

## 3. Discussion

Increasing evidence from basic and clinical investigations points to disruption of circadian rhythmicity in PD at both the behavioral and molecular level. Circadian dysfunction has been observed in PD patients and animal models [19], which might result in negative consequences for body homeostasis and could even exacerbate disease progression. Particularly, several studies have found alterations in sleep–wake cycles, visual performance, and hormone secretion patterns [19]. This is not surprising given the pivotal role that dopamine plays in circadian regulation as well as the role of circadian pathways in dopamine metabolism [48]. The molecular mechanisms linking circadian disruption and neurodegeneration are not fully understood yet. Considering mitochondrial function rhythmicity and the prominent role of metabolic dysfunction in PD, in the present study we focused on the interplay between circadian clock circuits and metabolism in parkin-null fibroblasts isolated from PD patients.

Considering that biological clocks tick and drive rhythmic cellular processes in central pacemaker cells as well as in peripheral tissues cells [49], first we demonstrated in synchronized NHDFs the occurrence of autonomous oscillatory mitochondrial respiratory activity synchronous with glycolytic activity. Our results suggest the occurrence of alternating phases in the overall cellular bioenergetics that undergo rhythmic changes between the energetic and quiescent state.

Of note, the observed rhythmic respiratory and glycolytic activities were apparently ultradian (i.e., occurring with a period of 12–15 h, shorter than the canonical 24 h of a circadian cycle). This feature was repeatedly observed and reported by our group in different synchronized cell cultures and irrespective of the protocol of synchronization utilized [29,30,50]. Although the rhythmic respiratory activity was proven to be linked to the circadian molecular clockwork, how it translates in ultradian cycling is not clear at this time. In other studies investigating the daily changes of the mitochondrial OCR activity, the measurements were reported only at two time points following synchronization [31]. Therefore, it is possible that features of the respiratory activity followed at short intervals for more than 24 h post-synchronization might have been missed. The only extended time-course of the OCR in synchronized cells published by a different group [51] reported a profile with “non canonical” circadian oscillations. Intriguingly, it has been reported that, following a quantitative mitochondrial proteomics analysis in mice, the rate-limiting enzymes in glucose and fatty acid oxidation (i.e., the pyruvate dehydrogenase complex and the carnitine palmitoyl transferase 1) underwent a daily *PER1/2*-dependent rhythmic oscillation but with phases displaced by about 12 h [52]. This observation was functionally confirmed by measurements of the OCR in purified mitochondria in the presence of either palmitoyl-CoA + carnitine or pyruvate + malate. The superimposition of the two harmonics would result in an ultradian cycling of the OCR. Likely, in our experimental conditions in intact cells with the mitochondrial respiration relying largely on endogenous substrates, a cell autonomous program which foresees preferential utilization of the two main respiratory substrates but with out-of-phase periods is unveiled.

Hence, we extended our analysis to fibroblasts with parkin mutation derived from two unrelated PD patients and fibroblasts from healthy subjects used as the control [10,11]. In non-synchronized PD fibroblasts we observed a moderate impairment of the respiratory and glycolytic fluxes, more severe in P1 fibroblasts, mainly occurring under stressed uncoupled respiration. This would explain the absence of significant changes in the mitochondrial membrane potential assessed under basic conditions between normal and patients fibroblasts. However, in P1 fibroblasts the presence of more fragmented mitochondria was observed.

When the same metabolic flux analysis was extended to synchronized cells, a completely different scenario appeared. As compared with normal fibroblasts behaving as NHDFs, both the PD fibroblasts (P1 and P2) displayed a dramatic damping in their time-dependent oscillatory profile of both respiratory and glycolytic activity. Similar results were attained using a completely different protocol of synchronization. For the first time, we demonstrated an alteration in the autonomous bioenergetic rhythmicity in fibroblasts obtained from PD patients with parkin mutation.

The energy-producing capacity of mitochondria is strongly related to their abundance, morphology, and dynamics, which depend on the processes of mitochondrial biogenesis, mitophagy, and fission/fusion, all shown to undergo circadian changes [40]. Parkin is a key protein factor in all these processes. Indeed, upon ubiquitylation of the transcriptional repressor parkin interacting substrate (PARIS) and its successive degradation, parkin facilitates the expression of the transcriptional co-activator PGC-1a, one of the major transcriptional regulators of mitochondrial biogenesis [53]. Moreover, the PINK1/parkin pathway promotes mitochondrial fission and/or inhibits fusion by negatively regulating mitofusins (MFN) and optic atrophy-1 (OPA1) function [12], and positively regulating dynamin-related protein 1 (DRP1) [54]. Finally, PINK1 and parkin act cooperatively in sensing and marking dysfunctional mitochondria in mammalian cells for their disposal via the mitophagy pathway [55].

As reported above, recent evidence highlighted the rhythmic patterns of the processes related to mitochondrial quality control [40]. In particular, it has been proven that PGC-1α is rhythmically expressed in several tissues [30,56]. Notably, PGC-1α interacts with the circadian clock machinery and REV-ERB-α increases PGC-1α activity through the AMPK-dependent activation of SIRT1 [57]. Conversely, PGC-1α has been shown to affect the functioning of the core clock machinery by increasing transcription of BMAL1 and CLOCK via interaction with RORα and RORβ [56]. The existence of a crosstalk between the clockwork machinery and the mitochondrial network has been proven that maintains bioenergetic homeostasis in response to circadian metabolic changes [31]. A key mediator of mitochondrial fission, DRP1, undergoes circadian regulation and this results in cycles of fission and fusion that are essential for circadian oscillations in ATP production [41].

Considering the role that parkin has in regulating these processes, it is not surprising that its mutation affects the mitochondrial respiratory functions. Parkin itself undergoes circadian oscillations in its expression, as we demonstrate here for the first time to the best of our knowledge in synchronized control fibroblasts. The expression of PINK1, that acts cooperatively with parkin in regulating mitochondrial quality control, showed an oscillatory profile as well, confirming previous results [31]. Conversely the PD fibroblasts showed an altered PINK1 rhythmic pattern, suggesting that parkin mutation interferes with the circadian nature of the mitochondrial dynamics/mitophagy.

When we analyzed the expression profile of *PGC-1α*, marked changes were observed both in its basic expression and its oscillatory profile in synchronized PD fibroblasts. This resulted most likely from deregulation of SIRT1, an NAD^+^-dependent histone/protein deacetylase, whose oscillatory pattern was markedly altered as well. PGC-1α activity is positively regulated through SIRT1-mediated deacetylation, among other modifications [58]. Interestingly, SIRT1 is involved in regulating the expression amplitude of several core clock genes, including *BMAL1, ROR-γ, PER2,* and *CRY1* [59].

Analysis of the expression of the core clock gene *ARNTL/BMAL1* resulted in similar oscillatory profiles both in synchronized normal and PD fibroblasts. This result is in contrast with previous in vivo studies showing a lower time-dependent level of *BMAL1* expression in PD patients and in a PD animal model. In any case, it should be noted that in the studies performed in humans, clock gene expression was evaluated on different peripheral blood leukocytes harvested at regular time intervals during the day [27], and in the study performed in a rotenone-induced PD male Wistar rat model [60], a deficit of dopamine possibly accounted for the different attained results. When we assessed the expression of the BMAL1 partner, *CLOCK*, a different scenario appeared. The synchronized PD fibroblasts displayed, indeed, a dramatic damping in the oscillatory expression of *CLOCK*. CLOCK is a histone acetyltransferase that is required for the circadian expression of many genes (among others *BMAL1* and *PER2*) that display circadian acetylation cycles [61,62]. BMAL1 acetylation promotes repression of its transcriptional activity [62]. The lack of *CLOCK* oscillatory expression, as well as the lack of its acetylation activity, can result in altered amplitude and/or acrophase of BMAL1/CLOCK heterodimer target genes belonging to the basic loop (*PER1–3*, *CRY1–2*) or to the auxiliary loop (*NR1D1*), as displayed in PD fibroblasts. Interestingly, NR1D1 is a critical regulator of neuro-inflammation and mediates microglial activation [63]. This is remarkably important considering that chronic neuro-inflammation is one of the key players in PD pathophysiology [64].

We are aware that the in vitro synchronization protocols, though enabling discovery of autonomous circadian rhythms, nevertheless cannot mimic other external entraining conditions. This can account for somehow different values of the cycling parameters such as the amplitude and the period. Indeed, the expression level of *CLOCK*, which is constitutive in the suprachiasmatic nucleus but follows a circadian rhythm in peripheral tissues/organs (though with a period shorter than 24 h [65], here exhibited an oscillatory period of about 15 h. Nevertheless, in the context of this study, since a comparative analysis of normal and PD patient-derived fibroblasts was carried out under identical conditions, we are confident that the significant large differences observed, as in the case of the *CLOCK* gene expression, provide essential information of the role of parkin in the interplay between mitochondrial function and the cellular biological clockwork.

The CLOCK-dependent acetylation is counteracted by the NAD^+^-dependent SIRT1 function [66]. Given that cellular NAD+ levels are coupled to metabolic activity, the identification of SIRT1 as histone/protein deacetylase counteracting CLOCK acetyl-transferase activity provides a strong connection in the biological clock/metabolism interplay 30,67]. In the parkin-null context, this is quite relevant. Indeed, it was previously demonstrated that as compared with healthy fibroblasts, the basal level of NAD+ and the NAD+/NADH ratio were both significantly lower in PD patient fibroblasts and increasing the NAD+/NADH ratio by pharmacological treatment enhanced SIRT1 deacetylation activity [11].

The above-reported results highlight the role of parkin in controlling on one hand the energetic metabolism and on the other hand the coordinated expression of the circadian clockwork cogs, with the two processes reciprocally interplaying [29,30,67]. However, to translate these observations in the context of Parkinson’s disease, a more suitable cellular model is needed [68]. To this aim we generated iPSCs from the normal and PD patient-derived fibroblasts used in this study and then re-differentiated them to iNSCs as neurospheres. Parkin mutation did not interfere with the retro-differentiation of fibroblasts into iPSC, probably because retro-differentiation into a quiescent stemness phenotype is characterized by repression of mitochondrial oxidative metabolism [69,70,71]. In addition, the expression of the core clock genes was found to be down-regulated in iPSCs as well [72]. However, down-regulation of the expression of *PINK1*, *PGC1A*, *SIRT1, PER3,* and *NR1D1* was revealed in PD iPSCs as compared with normal iPSCs. Differentiation of iPSCs and iNSCs as neurospheres from spontaneously generated embryoid bodies was also successfully achieved. Neurogenesis is reported to require both bioenergetic and clock genes up-regulation [73,74,75]. Indeed, several lines of evidence indicate that in embryonic pluripotent stem cells, as well as in iPSCs, the clockwork machinery is down regulated and that it starts to be “operative” following commitment [72,74,76,77,78,79]. Intriguingly, it has been reported that circadian rhythms of metabolism precede clock gene expression in mouse embryonic stem cells following differentiation [80]. This further highlights the tight interplay between cellular bioenergetics and clock genes.

Consistently, following neuralization of iPSCs we observed a relative increase in the transcription of both the genes involved in mitochondrial turnover and in the majority of those encoding the clock machinery cogs. However, a comparison between normal and PD-derived iNSCs resulted in significant quantitative and qualitative differences, suggesting that the parkin mutation impacts on neurogenesis. An indirect clue is provided by the proliferation rates of iNSCs, which when derived from the PD patient resulted markedly restrained, thus suggesting a fault in the energy generating system needed to support cell growth.

It must be pointed out that, as clearly shown in this study, the assessment of gene expression in the context of non-synchronized cells might result in less obvious severe alterations of clock gene-mediated functions. Therefore, more systematic analyses in synchronized iPSCs and iNSCs will be needed to better define the underlying pathways linking circadian clock circuits and neurodegeneration, and these experiments are ongoing in our laboratories.

## 4. Materials and Methods

### 4.1. Skin Fibroblast Harvesting and Culture Conditions

Primary skin fibroblasts from two patients (P1 and P2) affected by early-onset PD, with different parkin compound heterozygous mutations (P1 with del exon2–3/del exon3 [10], and P2 with del exon7–9/Glu409X [11]) and from two healthy subjects, were obtained by explants from skin punch biopsy, after obtaining informed consent.

Adult normal human dermal fibroblasts, purchased from Lonza Walkersville Inc., (Walkersville, MD, USA) were utilized as a third healthy control. Cells were grown in high-glucose Dulbecco’s modified Eagle’s medium (DMEM) supplemented with 10% (*v*/*v*) fetal bovine serum (FBS), 1% (*v*/*v*) l-glutamine, and 1% (*v*/*v*) penicillin/streptomycin, at 37 °C in a humidified atmosphere of 5% CO_2_.

The serum shock-induced synchronization was performed as in [32]. Briefly, the medium of cultured cells was exchanged with serum-rich DMEM containing 50% FBS for 2 h and then the medium was replaced with serum-free DMEM. Alternatively, fibroblasts cells were synchronized by incubation with 10 μM forskolin (directly added to the culturing medium) for 15 min; thereafter forskolin was washed-out by replenishing cultured cells with DMEM (+10% FBS) [33]. The cells were harvested and assayed at the different time points post-synchronization indicated in the text/figures. For measurement of the metabolic fluxes by Seahorse (see ahead), distinct clusters of cell samples, plated in the multi-well cartridge, were subjected to the synchronization protocols following a 4-h out-of-phase time-schedule enabling assessment of all the samples in the same experimental session. Cell cultures were typically utilized at a passage number below 10–12 and at a confluence of 80–85%.

### 4.2. Seahorse Analysis

Oxygen consumption rate (OCR) and extra-cellular acidification rate (ECAR) were measured in adherent fibroblast cells with a XF96 Extracellular Flux Analyzer (Seahorse Bioscience, Billerica, MA, USA). For OCR analysis, after replacing the growth medium with 180 μL of bicarbonate-free DMEM supplemented with 10 mM glucose 2 mM l-glutamine and 1 mM sodium pyruvate pre-warmed at 37 °C, cells were preincubated for 45 min before starting the assay procedure. After measuring basal respiration, oligomycin (1 μM), carbonyl cyanide m-chlorophenylhydrazone (0.5 μM), and rotenone + antimycin A (1 μM + 1 μM) were injected into each well sequentially to assess respectively coupling of the respiratory chain, maximal and non-mitochondrial oxygen consumption. The values were normalized to protein content in each well, determined with BCA assay.

### 4.3. Laser Scanning Confocal Microscopy (LSCM) Live Cell Imaging of Mitochondrial Membrane Potential and Mitochondrial Morphology Analysis

Cells cultured at low density on fibronectin-coated 35-mm glass-bottom dishes were incubated for 20–30 min at 37 °C with 0.5 μM tetramethylrhodamine ethyl ester (TMRE) to monitor mitochondrial membrane potential (ΔΨm) (Invitrogen, Molecular probes^TM^, Carlsbad, CA, USA). Stained cells were washed with PBS and examined with a Nikon TE 2000 microscope (NIKON, Tokyo, Japan); images collected using a 60× objective (1.4 NA)) coupled to a Radiance 2100 dual-laser LSCM system (Bio-Rad, Hercules, CA, USA); TMRE red fluorescence was elicited by exciting with the He-Ne laser beam (λex 543 nm). Acquisition, storage, and analysis of data were performed with LaserSharp and LaserPix software from Bio-Rad or ImageJ version 1.37. Mitochondrial morphology was scored by software-based methods using ImageJ software and plugins (https://imagej.nih.gov/ij/). Images of the TMRE-stained cells were converted to binary (black and white) images by auto-thresholding, and mitochondrial particles were analyzed for area (a), perimeter (p), and circularity (c). Metrics that reliably reported the morphology of the mitochondrial network are the cumulative area:perimeter ratio (∑a/∑p) and the elongation factor (1/c = p^2^/(4π × a).The cumulative area:perimeter ratio (or interconnectivity factor) is computed as the summed particle area in a region of interest [81] divided by the summed particle perimeter (including the perimeter of enclosed spaces or “holes”). This metric is particularly effective at detecting the transition from elongated, isolated mitochondria to a reticular network of interconnected mitochondria. The elongation factor is reported as an average of all particles in a ROI, has a minimum value of 1 (for perfect circles), and captures well the transition from punctiform to elongated, complex-shaped, but still isolated mitochondria.

### 4.4. Real-Time PCR

The purification of total RNA from fibroblasts was carried out by using Aurum Total RNAMini Kit (Bio-Rad, Hercules, California, USA) according to the manufacturer’s protocol. 0.5 microgram of total RNA was then reverse-transcribed to generate cDNA for PCR by using the iScript cDNA Synthesis kit (Bio-Rad, Hercules, California, USA). Semi-quantitative determination of mRNA levels were performed by real-time qRT-PCR, using SYBR Green (Bio-Rad, Hercules, California, USA). Reactions were performed in duplicate for each sample. Multiple reactions were performed in a volume of 20 μL containing 10 μL of 2× PCR master mix, 1× of validated specific primers (Qiagen, Venlo, The Netherlands), and 2 μL of cDNA template. Amplifications were performed in the Stratagene MX3000P Real-Time PCR Detection System (Stratagene, San Diego, CA, USA), using the following cycle program: denaturation step at 95 °C for 10 min followed by 40 cycles of denaturation at 95 °C for 15 s, annealing at 55 °C for 1 min, and extension at 72 °C for 30 s. The relative mRNA expression levels were calculated by using the comparative CT method (ΔΔCT). Quantitative normalization for each sample was performed by using glyceraldehyde-3-phosphate dehydrogenase (GAPDH) as an internal control. Validated primers (Qiagen, Venlo, The Netherlands) for qRT-PCR are provided in Table S1.

### 4.5. Fibroblast Reprogramming

1 × 10^5^ skin fibroblasts were nucleofected with three episomal plasmids: pCXLE-hUL (Addgene #27080), pCXLE-hSK (Addgene #27078) and pCXLE-hOCT4-shp53 (Addgene #27077), and plated in fibroblast medium for one week. From day 8 the cells were cultured in NutristemXF medium (Biological Industries, Kibbutz Beit Haemek, Israel). iPSC colonies were manually cut and passaged for expansion. Absence of mycoplasma contamination was verified by PCR analysis using EZ-PCR kit (Biological Industries, Kibbutz Beit Haemek, Israel).

### 4.6. Immunofluorescence Staining

Cells were fixed using 4% paraformaldehyde. Blocking buffer (PBS, 20% Normal Goat Serum, 0.1% Triton X-100) was incubated for 30 min at room temperature. Primary antibodies were incubated overnight at 4 °C. Secondary antibodies in PBS 1× were added and left for 1 h at room temperature. Nuclei were counterstained with DAPI. Micrographs were taken using a Nikon C2 fluorescence microscope and NIS Elements 1.49 software. 

### 4.7. In Vitro Spontaneous Differentiation

The cell clumps from iPSCs were plated on Petri dishes in NutristemXF medium. One day later, NutristemXF medium was substituted with in differentiation medium: DMEM/F12, 20% KSR (Gibco), 0.1 mM NEAA, 0.1 mM β-mercaptoethanol, 1% Pen/Strep. The embryoid bodies (EBs) were grown for two weeks.

### 4.8. Karyotyping

Karyotype analysis was carried out on GTG-banded metaphases. Cells were treated with a 0.1 µg/mL COLCEMID solution. Metaphases were obtained by adding 30 mM KCl in 10% FBS at 37 °C for 6 min and by fixation, using a 3:1 ethanol:acetic acid solution. Fifteen metaphases were counted and three karyotypes analyzed.

### 4.9. Teratoma Formation

Approximately 3 × 10^5^ dispase-treated iPSCs, in 100 µL of Matrigel, were injected into the right flank of nude mice following ethical guidelines. Teratomas were dissected, fixed in formalin, paraffin-embedded, sectioned, and stained with hematoxylin/eosin. The presence of differentiated tissues representative of the three embryonic germ layers was analyzed.

### 4.10. iPSC Neuralization

iPSC-derived neural stem cells (iNSCs) were obtained using a published protocol [47].

### 4.11. Protein Measurement

Total protein concentration was determined by the BCA protein assay, using bovine serum albumin as the standard.

### 4.12. Cosinor Analysis

The following modified COSINOR equation was used to fit the experimental data in synchronized cells:

f(t) = M + A∙e^(t∙a)^∙cos(2π∙(t-φ)/P) + s∙t; (1)

where M = mesor, A = amplitude, a = attenuation factor of A, φ = acrophase, P = period, and s = slope. The best fit was attained using the software GraFit (V 4.0.13, Erithacus Software Limited, East Grinstead, West Sussex, UK) by manual insertion of ranges of initial parameters.

### 4.13. Statistical Analysis

Data are shown as mean ± SEM. Data were compared by an unpaired Student’s *t*-test. Differences were considered statistically significant when the *p*-value was less than 0.05. All analyses were performed using Graph Pad Prism (Graph Pad software, v 6.01, San Diego, CA, USA).

## 5. Conclusions

The major and novel findings of this study unveil that parkin mutations dramatically impact on cell autonomous rhythmic processes, such as metabolic fluxes and functioning of the clockwork machinery. Most notably, the differences observed in vitro between normal and mutated fibroblasts are particularly evident upon cell synchronization; otherwise they result faint. In addition, preliminary results suggest impaired neurogenesis in iPSCs obtained by retro-differentiation of PD patient fibroblasts with parkin mutations. Further investigations will be needed to better define the molecular mechanisms underlying the interplay between mitochondrial energy metabolism, circadian clock circuits, and neurodegenerative disease pathophysiology, but our results suggest, for the first time, a novel role for parkin at a hitherto unappreciated level of complexity in the process of neurodegeneration, providing new perspectives for therapeutic interventions.

## Supplementary Materials

Supplementary materials can be found at https://www.mdpi.com/1422-0067/20/11/2772/s1.

## Abbreviations

BMAL1/ARNTL	aryl hydrocarbon receptor nuclear translocator like
CLOCK	circadian locomotor cycle kaput
CRY1,2	cryptochrome circadian regulator 1 and 2
NR1D1	nuclear receptor subfamily 1 group D member 1
iPSC	induced pluripotent stem cells
iNSC	induced neural stem cells
NHDF	normal human dermal fibroblasts
PER 1,2,3	period circadian clock 1,2,3
PGC-1α	peroxisome proliferator-activated receptor (PPAR)-γ coactivator 1 alpha
PD	Parkinson’s disease
SIRT1	sirtuin 1
