# Supplementary material for: Parkin Mutation Affects Clock Gene-Dependent Energy Metabolism
Source: Int J Mol Sci. 2019 Jun 5;20(11):2772. doi: 10.3390/ijms20112772 (PMC6600341; doi:10.3390/ijms20112772)
Supplement: Supplementary file 1 [file ijms-20-02772-s001.pdf]

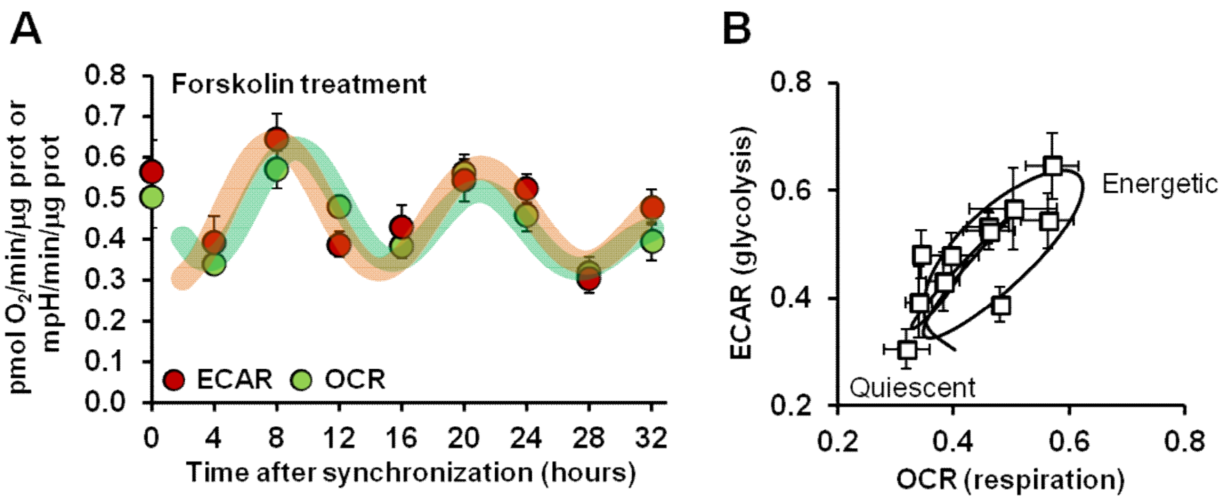

Figure S1. Analysis of mitochondrial respiration and glycolysis in forskolin-treated synchronized NHDF cells. (**A**) The oxygen consumption rate (OCR) and extracellular acidification rate (ECAR), were assessed by Seahorse analyzer in intact NHDF every four hours post-synchronization for 36 h under resting conditions. Data are expressed as pmol O_2/min/µg protein for OCR and mpH/min/µg protein for ECAR and are means ± SEM of three independent measurements carried out in three technical replicates under each conditions. See Materials and Methods for experimental details. (**B**) Energy map obtained plotting the ECAR and OCR values obtained in (**A**).

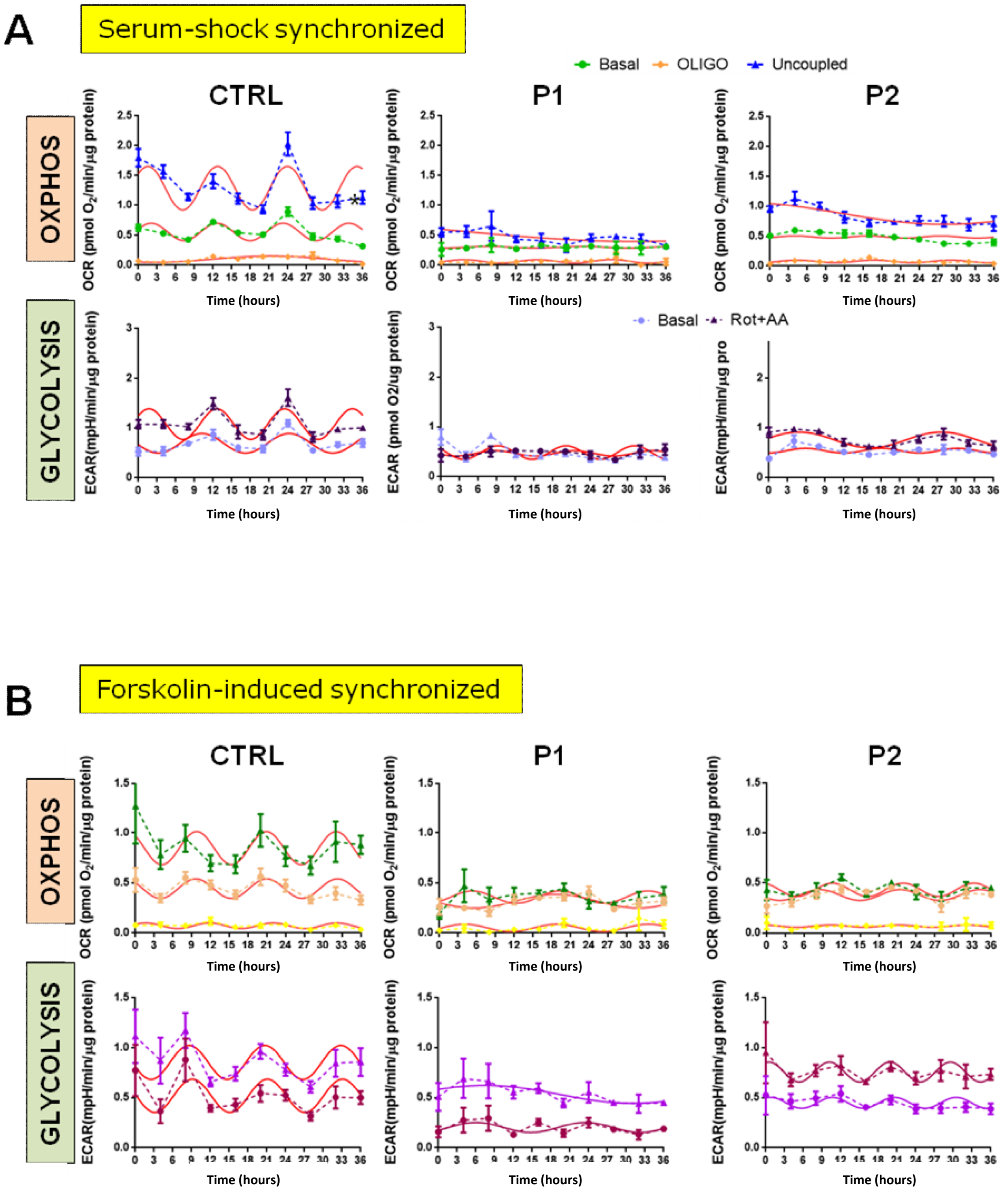

Figure S2. Measurement of the mitochondrial respiratory and glycolytic activity in intact fibroblasts synchronized by serum shock (**A**) or forskolin pre-treatment (**B**). The graphs display for normal fibroblasts (CTRL) and PD-patients fibroblasts (P1 and P2) the OCR-OXPHOS and ECAR-Glycolysis under different conditions as follows: basal, in the presence of oligomycin, in the presence of FCCP (uncoupled) for OCR; basal, in the presence of the respiratory chain inhibitors rotenone + antimycin A (Rot + AA) for ECAR. The time points were fitted with the COSINOR function (continuous curve-lines). The values shown are means ± SEM of three independent measurements carried out in three technical replicates under each conditions. See Materials and Method for details.

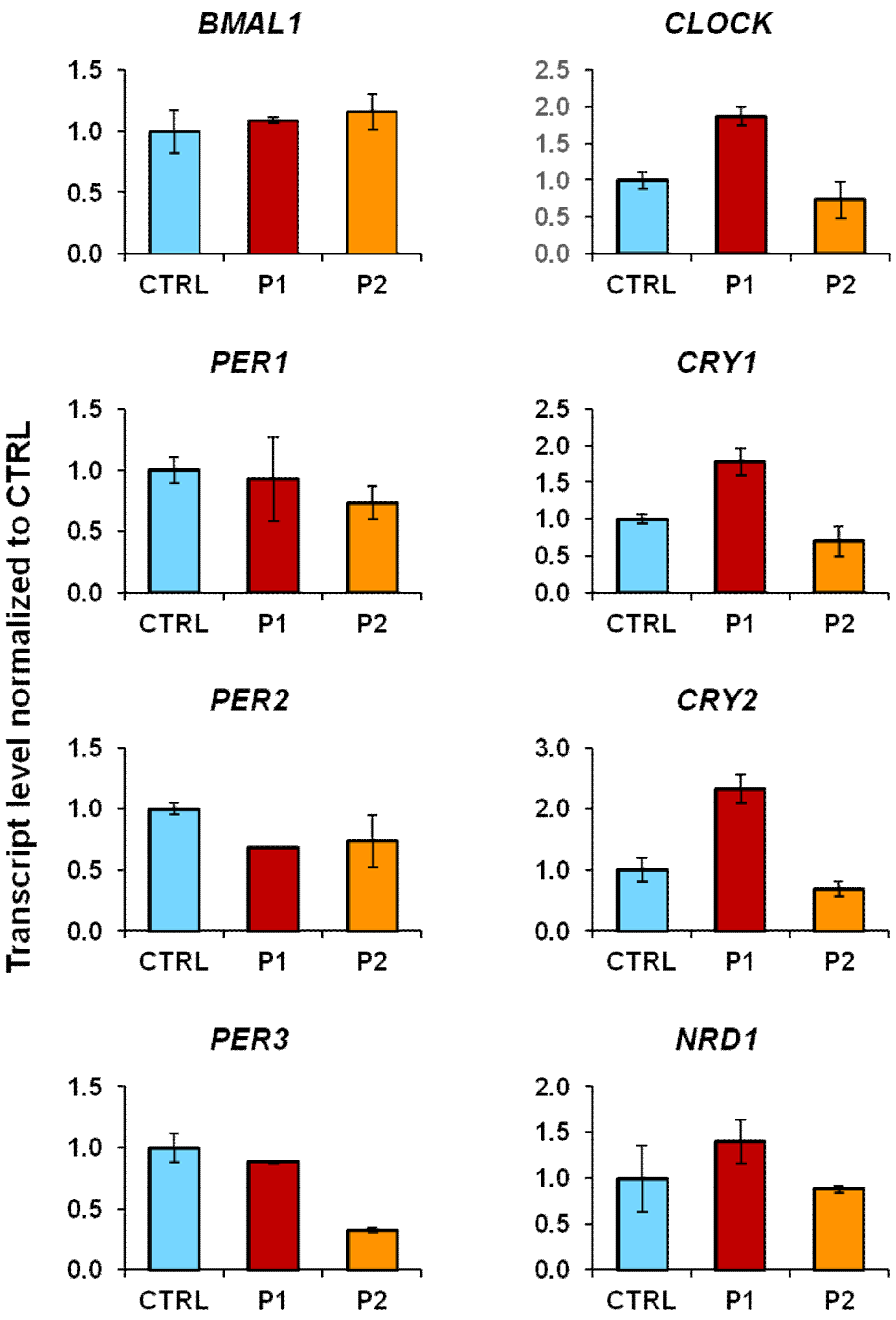

Figure S3. Transcription levels of core clock genes in normal (CTRL) and PD-derived (P1, P2) fibroblasts. The mRNA was isolated from non-synchronized fibroblasts, subjected to qRT-PCR analysis and the values normalized to the housekeeping gene GAPDH and to CTRL. The bar-values are means ± SEM of two independent measurements carried out in three technical replicates under each conditions. See Materials and Methods for experimental details.

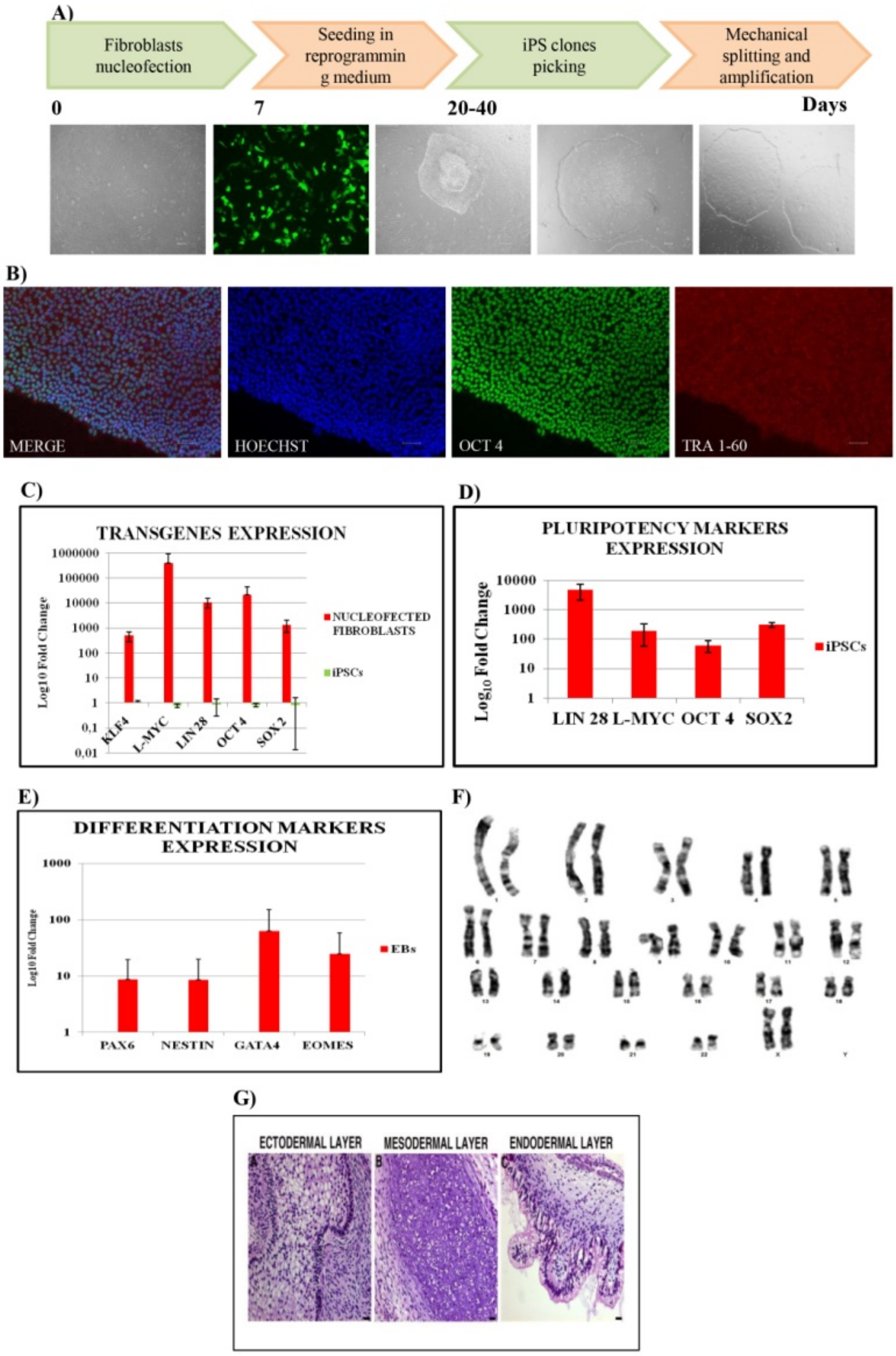

Figure S4. Parkinson induced Pluripotent Stem Cell Production. Cells were reprogrammed under virus-free and feeder-free conditions, in presence of a xeno-free medium. The efficiency of nucleofection was measured through GFP positive cells (**A**). After three weeks from nucleofection, some clones appeared on reprogramming plate, and each of them was picked up and amplified.(**A**) The chosen iPSC lines showed the typical human stem cell-like morphology with well-defined and flattened cell colonies. The objective magnification was 10× (**A**). After ten passages, iPSC colonies did not express transgenes; the RNA extracted from nucleofected fibroblasts was used as a positive control of transgene expression (**B**). A quantitative real-time PCR (qPCR) demonstrated expression levels of five pluripotent markers: *OCT4*, *LIN28*, *L-MYC*, *SOX2*. Total RNA was isolated from cultured cells with Trizol (Life Technologies). Reverse transcription of 1µg of RNA was performed using High Capacity cDNA Reverse Transcription Kit (Applied Biosystems) after digestion with DNAse I (Life Technologies). Each RNA sample was tested in duplicate, 18S was used to normalize transcript abundance and calculations were performed with the delta Ct method. Statistical

analyses were performed on three independent experiments. (**C**). The iPSC line produced embryoid bodies which spontaneously differentiated into three germ layers expressing the ectodermal markers NESTIN and PAX6, the mesodermal marker EOMES and the endodermal gene GATA4 (**D**). Pluripotency of this cell line was also confirmed by the expression of transcription factor OCT4 and of surface marker TRA-1-60 obtained through immunofluorescence analysis. the objective magnification was 10× (**E**). Differentiation capacity into three germ layers was also demonstrated through in vivo teratoma formation (**F**). Karyotype analysis demonstrated no accumulated chromosomal aberrations. The objective magnification was 5× (**G**).

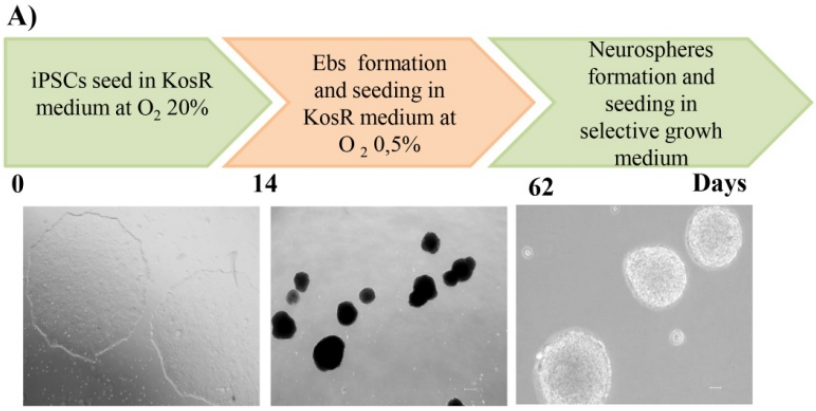

Figure S5. iPS-neuralization. Schematic diagram illustrating the overall strategy to generate hiNSCs from hiPSCs. Phase contrast images illustrate the typical morphology of cells at each stage. The objective magnification was 10×.

Table S1. Primers list.

Gene Name	Product Number
BMAL1	PAHS-135Z
CLOCK	PPH06233A
NR1D1	PPH02259A
PER1	PPH02075A
PER2	PPH06234A
PER3	PPH19810B
CRY1	PPH06231A
CRY2	PPH06235A
PARK2	PPH05959A
PINK1	PPH20890B
PPARGC1A	PPH00461F
SIRT 1	PPH02188A
GAPDH	PPH00150F
